# Correlations between noninvasive tests as predictors of liver fibrosis and of viral response in chronic hepatitis C patients

**DOI:** 10.1186/1471-2334-13-S1-P54

**Published:** 2013-12-16

**Authors:** Oana Streinu-Cercel, Elena-Sînziana Daia, Mihail Tiberiu Daia, Anca Streinu-Cercel, Tania Gudu, Adrian Streinu-Cercel

**Affiliations:** 1 National Institute for Infectious Diseases “Prof. Dr. Matei Balş”, Bucharest, Romania; 2 Carol Davila University of Medicine and Pharmacy, Bucharest, Romania; 3 Internal Medicine and Rheumatology Department, “Sf. Maria” Clinical Hospital, Bucharest, Romania

## Background

The noninvasive assessment of liver fibrosis in chronic hepatitis C patients is very important as it dictates the disease prognosis and the indication for therapy, narrowing down the use of the liver biopsy. Standardized scores (FibroTest, FibroMax) and imaging techniques (FibroScan) have been validated in this respect. As for the noninvasive prediction of the sustained viral response (SVR), GenoFibroTest and Prometheus are two indexes that may be used. This study's aim is to compare several biological scores with these standardized scores for evaluating fibrosis and the two indexes for prediction of SVR.

## Methods

We performed a cross-sectional analysis on a group of patients with chronic hepatitis C evaluated at the National Institute for Infectious Diseases “Prof. Dr. Matei Balş”. We assessed serum tests, FibroScan, FibroTest and IL28B polymorphism. We calculated several biological scores (APRI, Lok, Forn’s, FIB-4 and Bonacini) and compared them with each other and with FibroScan/FibroTest and we calculated and compared the GenoFibroTest and Prometheus indexes, verifying the statistic correlation of the results.

## Results

The study included 56 patients (of which 26 were males); the median age was 52±12 years. The median necroinflammatory activity was 0.58±0.25, as evaluated through ActiTest. The median FibroTest values were 0.55±0.30 while the median FibroScan scores were 8.8±9.7 kPa. The areas under the receiver operating characteristic (AUROC) for APRI, Lok, Forn’s, FIB-4, Bonacini scores and FibroScan, compared to FibroTest were 0.70, 0.78, 0.85, 0.80, 0.78 and 0.71 respectively, for F≥2; and 0.83, 0.79, 0.92, 0.86, 0.77 and 0.77, respectively, for F=4. As for the prediction of SVR, for a cut-off of GenoFibroTest of 0.4, Prometheus index’s AUROC was 0.85.

## Conclusion

All the evaluated scores had excellent predictive value, with Forn’s being the best biological score, especially for the assessment of significant fibrosis (F2-F4). Prometheus index had a good predictive value of the patients that could benefit only from double therapy, in the context of the triple association. If more non-invasive tests could be standardized, the stage of fibrosis and the SVR could be easily estimated, and the need for biopsy could be narrowed down.

